# Nature inspired optimization tools for SVMs - NIOTS

**DOI:** 10.1016/j.mex.2021.101574

**Published:** 2021-11-02

**Authors:** Carlos Eduardo da Silva Santos, Leandro dos Santos Coelho, Carlos Humberto Llanos

**Affiliations:** a Universidade de Brasília – UnB, Brasília - DF - Brasil; b Instituto Federal de Educação, Ciência e Tecnologia do Tocantins - IFTO, Palmas - TO - Brasil; c Pontífica Universidade Católica do Paraná - PUC- Curitiba - PR, Brasil

**Keywords:** Support vectors machines, Parameters selection problem, Adaptive parameters control, Differential evolution algorithm, Multi-objective optimization problem

## Abstract

Support Vector Machines (SVMs) technique for achieving classifiers and regressors. However, to obtain models with high accuracy and low complexity, it is necessary to define the kernel parameters as well as the parameters of the training model, which are called hyperparameters. The challenge of defining the more suitable value to hyperparameters is called the Parameter Selection Problem (PSP). However, minimizing the complexity and maximizing the generalization capacity of the SVMs are conflicting criteria. Therefore, we propose the Nature Inspired Optimization Tools for SVMs (NIOTS) that offers a method to automate the search process for the best possible solution for the PSP, allowing the user to quickly obtain several sets of good solutions and choose the one most appropriate for his specific problem.

• The PSP has been modeled as a Multiobjective Optimization Problem (MOP) with two objectives: (1) good precision and (2) low complexity (low number of support vectors).
• The user can evaluate multiple solutions included in the Pareto front, in terms of precision and low complexity of the model.
• Apart from the Adaptive Parameter with Mutant Tournament Multiobjective Differential Evolution (APMT-MODE), the user can choose other metaheuristics and also among several kernel options.

## Graphical Abstract

**Figure:**
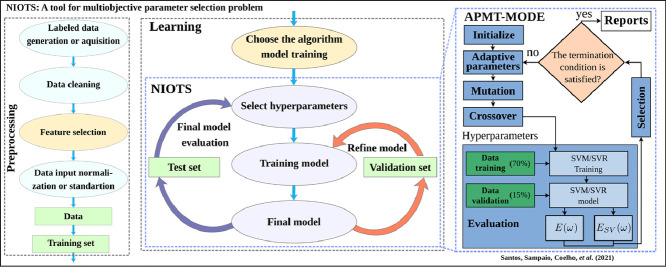

Specification table

**Table:** 

Subject Area	Computer Science
More specific subject area	Support Vector Machines (SVMs), Machine Learning, and Pattern recognition.
Method name	NIOTS: A flexible method for obtaining hyperparameters for SVMs via nature inspired metaheuristics, giving flexibility to the user in choosing the kernel, the metaheuristic to be used and the result that best suits the specific problem.
Name and reference of original method	Multi-objective adaptive differential evolution for SVM/SVR hyperparameters selection (*DOI**10.1016/j.patcog.2020.107649*).
Resource availability	https://github.com/profcarlosifto/NIOTS.git

The development of models based on Machine Learning (ML) is trendy due to their ability to generate efficient models from a set of data [5]. The ML techniques are subdivided into two large groups depending on the available data, namely, supervised and unsupervised ones. Our proposed method is based on a multiobjective metaheuristic optimization approach to select Support Vector Machines and Support Vector Regressor (SVM/SVR's) hyperparameters such as described in [11].

In this way, we present a method for solving the Parameter Selection Problem (PSP) for both SVM/SVR that uses supervised training. In this case, we seek to reach an optimal set of hyperparameters to minimize the number of support vectors and maximize generalization capacity. A significant problem is that SVM/SVR requires choosing a specific kernel to work with a particular dataset. In this way, the PSP consists of both obtaining the model parameters *C* (regularization parameter) for SVMs and the ∈-insensitive tube width and *C* for SVR, apart from the parameters for the chosen kernel. Both sets of parameters together (SVM/SVR and kernel) are denoted as *hyperparameter sets*.

For solving the PSP we have created several steps and strategies into the Nature-Inspired Optimization Tool for SVM/SVR (NIOTS), which was developed to automate the definition of SVM/SVR hyperparameters, seeking a balance between complexity and accuracy. Thus, the PSP is treated as a multi-objective optimization to minimize both the empirical risk and the complexity, which are contradictory criteria models. Apart from the NIOTS automating the solution of PSP, the same allows the user to select a series of steps for each target problem, choosing the metaheuristics to be used, the kernel type, and generating a Pareto front to be analyzed. Once the user chooses among a set of options, NIOTS generates a report containing the Pareto front and Pareto set. Bearing in mind that the NIOTs allows us to choose from a tree of possibilities, our method empowers the designer to make the most appropriate decision for his problem, comparing the results of each possibility offered by NIOTS.

SVMs were developed by Vapnik [3]. The training of SVMs consists of solving a convex quadratic optimization problem, making it possible to use algorithms based on a gradient to obtain the optimal solution [2], for instance, the algorithm Sequential Minimal Optimization (SMO). The training process of an SVM is a convex quadratic optimization problem, as shown by Eq. 1, which is a necessary and sufficient condition to guarantee a global optimal solution.

(1) $$\begin{array}{c} \max\;L_{c}\left(\alpha_{i}\right)=\sum_{i=1}^{N}\alpha_{i}-\frac{1}{2}\sum_{i=1}^{N}\sum_{j=1}^{N}\alpha_{i}\alpha_{j}y_{i}y_{j}K\left(x_{i},\;x_{j}\right)\\ s.t.\\ \;\sum_{i=1}^{N}\alpha_{i}y_{i}=0,\;\;\;\;\;0\leq \alpha_{i}\leq C,\;\;i\;=\;1,\;.\;.\;.,\;N,\;\xi_{i}\geq 0, \end{array}$$

$$\xi_{i}\geq 0.\left(\mathrm{This}\mathrm{equa}\mathrm{tion}\mathrm{was}\mathrm{join}\mathrm{ed}\mathrm{in}\mathrm{equa}\mathrm{tion}\left(1\right)\right)$$

where *C* is a regularization parameter, *N* is the training set cardinality, $\alpha^{\prime }s\;$ are the Lagrange multipliers, $\;\xi_{i}\;$are the slack variables and *K* (.) is the symmetric Positive Semidefinite (PSD) matrix. This matrix must satisfy Merce's conditions and is called *kernel matrix* or *kernel function* [4].

The optimal solution to the optimization problem of Eq. 1 is a sparse vector of weights α (the Lagrangian coefficients) associated with each training set element (see Eq. 2).

(2) $$\hat{f}\left(x\right)=\sum_{i=1}^{\#\mathrm{SV}}\alpha_{i}K\left(x,\;x_{i}\right)+\rho$$

where $\hat{f}\left(x\right)\;$is the predict value of the vector input x, *#SV* is the support vectors set cardinality, $\alpha_{i}\;$ are the Lagrangian coefficients, $K(x,\;x_{i})\;$ is the kernel function, $x_{i}$ is the *i-*th training vector and ρ is the bias. Thus, the greater the sparsity of the optimal vector, the lower the complexity of the SVMs. Therefore, we seek SVM/SVRs with the least complexity possible and with the greatest accuracy. Both complexity and accuracy of an SVM are controlled indirectly by the *C* (see Eq. 1) and the kernel parameters (see Table 1), which together are called hyperparameters of the model. The suitable choice of hyperparameters is strongly related to implicit characteristics to each set of training data and the chosen kernel, requiring the designer to handle hyperparameters and considerable effort in training models with different parameters.

**Table 1:** Kernel parameters.

Kernel	Kernel function	Parameters
Polinomial	$K\left(x,y\right)={\left(\beta x\;\cdot y+\;\kappa \right)}^{d}$	β,κ and d
Gaussian	$K\left(x,y\right)=exp\left(-\frac{∥x-y|{|}^{2}}{\gamma }\right)\;$	γ
Arc cosine (Cho e Saul 2009)	$K\left(x,y\right)=1-\frac{1}{\pi }\;\mathrm{co}s^{-1}\left(\frac{x\;\cdot \;y}{∥x∥∥y∥}\right)$	none
Cauchy (Drewnik e Pasternak-Winiarski 2017)	$K\left(x,y\right)=1/\left(1+\frac{∥x-y|{|}^{2}}{\lambda^{2}}\right)$	λ

Fig. 1 shows a flow chart diagram of the steps to be followed to obtain models based on machine learning. The steps (a) *choice of training technique*, (b) *features selection*, and (c) *hyperparameters selection* deserve to be highlighted because they consist of complex problems and a low-quality solution compromise the final model's performance [1]. Due to these problems' complexity, it is common to use metaheuristics to find solutions.

**Fig. 1:**
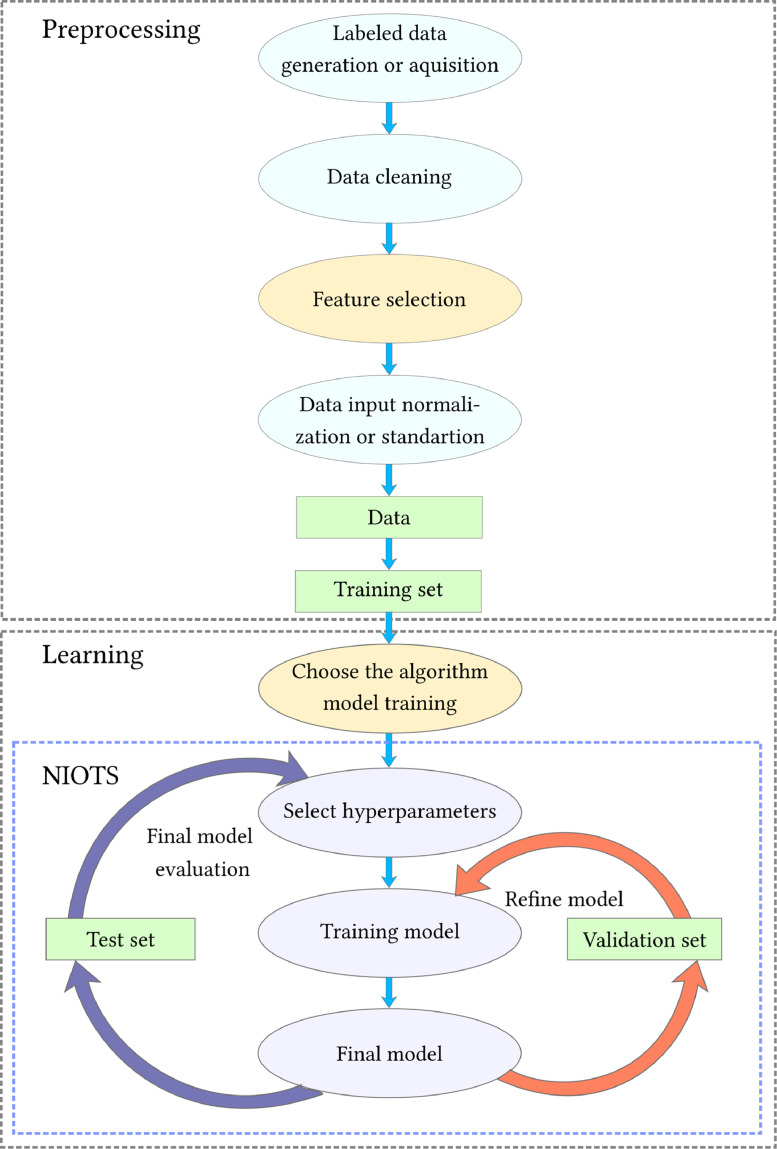
Machine learning flowchart.

To develop a data-based model the data must be prepared. The initial set of steps we call the pre-processing block, as shown in Fig. 1. Pre-processing consists of the following steps: (a) data acquisition or generation; (b) data cleaning; (c) selection of characteristics; (d) normalization or standardization; and (e) dividing the data into three sets, e.g., training, validation, and testing. In preprocessing we highlight step (e), which is a complex problem and of fundamental importance for the final result of the model in question [8].

From the data sets obtained in the pre-processing block, there are the tasks included in the *learning block* (details in Fig. 1), which consist of the following steps: (a) *choice of the training algorithm,* (b) *selection of hyperparameters*, (c) *training the model* and, (d) *final evaluation*. In this block all steps are critical.

Within the learning box, there is a blue NIOTS *sub-block* that selects SVM hyperparameters iterating over the model refinement cycle. The available metaheuristics in the tool e.g., (1) Adaptive Parameters - Multi-objective Differential Evolution (AP-MODE), (2) Adaptive Parameters with Mutation Tournament - Multi-objective Differential Evolution (APMT-MODE), and (3) Multi-objective Particle Swarm Optimization (MOPSO) allow evaluating the models with the data from the validation set through the metrics ACC (Accuracy) or MSE (Mean Squared Error), as well as the complexity of the model (support vector set cardinality -#SV). This strategy produces, as a result, a report with the set of solutions non-dominant, that is, the best models according to the criteria of accuracy and complexity.

The MOPSO employs the basic Particle Swarm Optimization (PSO) designed by Kennedy, [10] to update particles' velocity and position along the optimization process. We pick the global best particle and individual best particle from a non-dominated set with the same technique as in Santos [11]. In addition, the user has to choose the initial and final inertia, social and cognitive coefficient, the particle max velocity, and the population size.

In our proposal, NIOTS (see Fig. 1) automates the steps in purples (into the blue square), where the metaheuristics act over *Refine model* cycle (red arrows) and, as well as in the evaluation of the *Final models* (*Final model evaluatio*n cycle, see purple arrows). Usually, the metaheuristics have one metric to address for a solution, but this strategy induces overfitting. Therefore, to find the balance between generalization capacity and complexity, we model the PSP as a multi-objective problem taking as objective functions the support vectors cardinality (#SV) and the accuracy (ACC/MSE).

The three metaheuristics available in the NIOTS (e.g., APMT-MODE, AP-MODE, and the MOPSO) were coded in *m* language (from Matlab), offering to the user a Graphical User Interface Development Environment (GUIDE). In this way, our system has four tabs: (a) *Optimization*, (b) *Ensembles*, (c) *Prediction*, for testing the generated models and, (d) *Statistics*, for evaluating the results via statistical significance tests (see Fig. 2). Besides, NIOTS offers the user a certain degree of flexibility by the possibility to choose among several possible paths through the options described in Table 2.

**Fig. 2:**
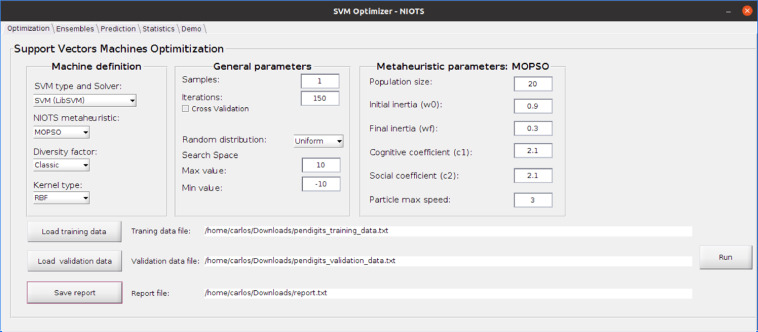
NIOTS optimization interface.

**Table 2:** NIOTS description options on optimization panel

Panel	Options	Descriptions
Machine definition	Model	SVM/LibSVM
		SVR/LiBSVM
		Grid Search SVR
		Grid Search SVM
	Optimization Algorithm	MOPSO
		APMT-MODE
	Kernel	RBF
		Polynomial
		Acos
		Cauchy
General parameters	Samples	Independent experiments amount.
	Iterations	Optimization algorithm iterations amount.
	Cross-validation	Enable the cross-validation training/validation process.
	Random distribution	Uniform
		Normal
		Cauchy
	Search space	The *C* and γ upper bounds
		The *C* and γ lower bounds
		The ε-insensitive tube upper bound
Metaheuristics parameters: MOPSO	The swarm size	The swarm particles amount
	Initial inertia	The PSO Initial inertia factor
	Final inertia	The PSO Final inertia factor
	Cognitive coefficient	The particle confidence level is in its own best position.
	Social coefficient	The particle confidence level is the best particle position overall.
	The particle max speed	The particle max speed allowed by PSO.
Metaheuristic parameters: AP/APMT-MODE	Population size	The Differential Evolution (DE) individuals amount.
	Scale factor	Initial scale factor.
	Crossover rate	Initial crossover rate.

In the NIOTS optimization environment, it is possible to optimize the hyperparameters of classifiers and regressors, using the MOPSO and APMT-MODE metaheuristic to solve the parameter selection problem formulated as a MOOP. The solutions obtained by the MOPSO and APMT-MODE metaheuristics are the suitable hyperparameters for the SVM/SVR.

The graphical interface of NIOTS (see Fig. 2) was developed to facilitate the several configuration possibilities that the system provides. The optimization environment is split into three panels: (1) Machine Definition, (2) General Parameters, and (3) Algorithm Parameters. Table 2 presents the description of each of the panel options.

In addition to the panels described in Table 2, the bottom has three buttons. The l*oad-training-data* button reads the file containing the training data, as shown in Fig. 3. To load the validation data, the *load-validation-data* button is used. This option is available if the *cross-validation* [9] option is not active. Both the name and location where the reports will be saved are chosen using the *save-report* button, and the *run* button starts the optimization process.

**Fig. 3:**
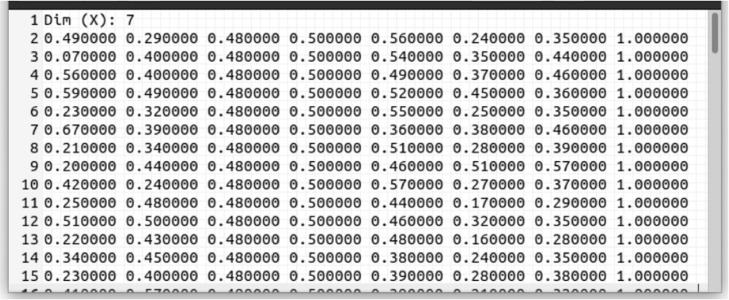
NIOTS input file example

The AP/APMT-MODE parameters do not appear in Fig. 2, because only parameters of metaheuristic chosen in the *Machine definition* panel are displayed. This behavior of the program prevents the user from confusing the metaheuristics parameters.

The LiBSVM (a Library for Support Vector Machines) tool (Chang, 2011) conducts the training using the APMT-MODE or MOPSO multi-objective metaheuristics for obtaining the optimized hyperparameters. These models are evaluated with the data from the validation set, returning the metrics of the model (generalization capacity and complexity). In Fig. 1 this is represented by the *refine-model* cycle.

In the flowchart of Fig. 4, the variable ω is the vector of the hyperparameters and the functional values *E*(ω) and *C_SV_*(ω) are the metrics of generalization capacity and complexity, respectively. The filled arrows, in black, represent the data flow between the blocks, while the white arrows indicate the possible options that the user can adopt, also presented in Table 2.

**Fig. 4:**
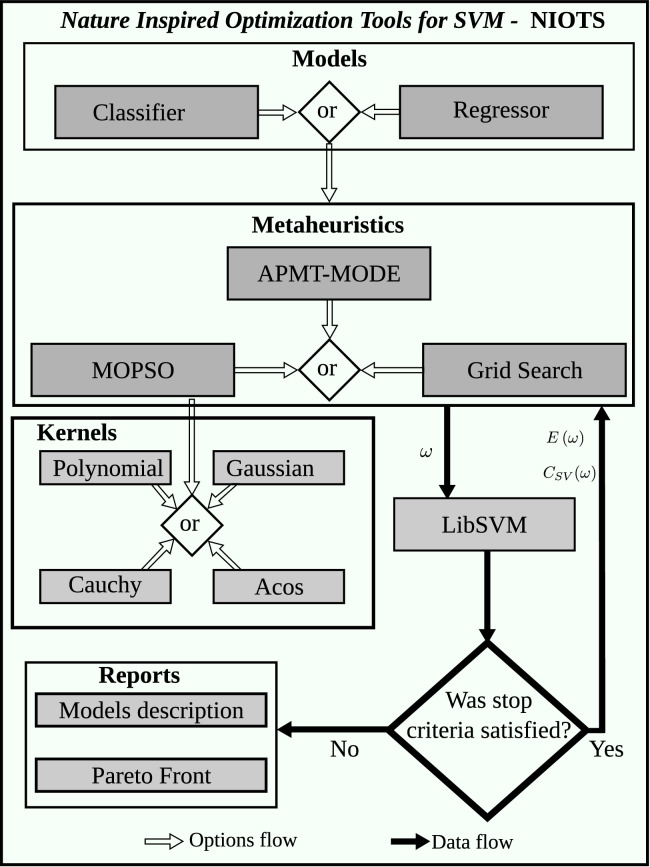
NIOTS flowchart options and dataflow

At the end of the optimization process, three types of reports are generated, represented in Fig. 2 by the *reports file* block. For each hyperparameter of the Pareto Front (PF) set (in details Fig. 4), a report file is generated, called *model-data*, which has all the data necessary to implement the model in question. Fig. 5 is an example of this report.

**Fig. 5:**
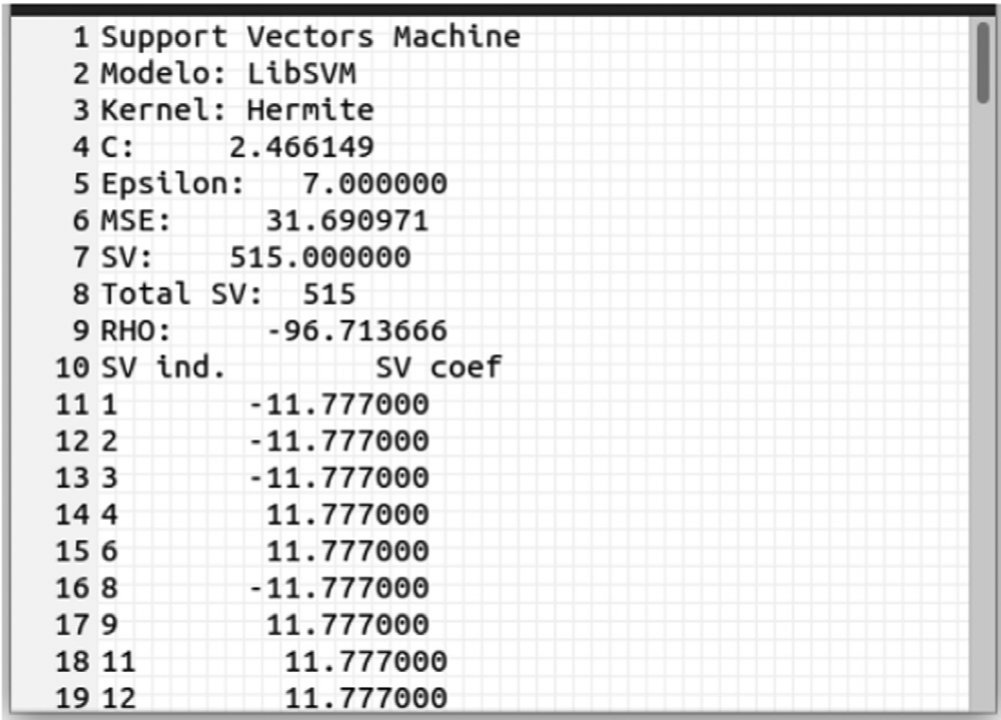
The models report file

The Pareto Front report (details in Fig. 6) contains the user-defined AP/APMT-MODE/MOPSO parameters and the elements of the Pareto Set where the index column represents each element of the set, which is composed of obtained hyperparameter (*C*, order, and ε) for an Hermite polynomial kernel, as well as their respective PF objectives (MSE and SV columns).

**Fig. 6:**
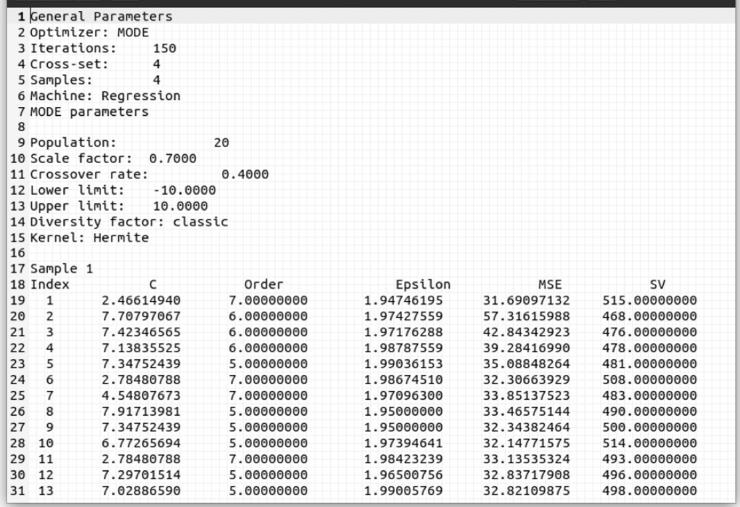
NIOTS report file for SVR, first three columns are Pareto set and the two last one are Pareto front

In the SVM/SVR design, a kernel must be defined as a priori, which has several parameters. Due to their particular characteristics, each kernel combined with each benchmark generates a different PSP. Each combination of kernel and training set generates a distinct optimization problem with several local minima, making choosing the optimal hyperparameters a complex task. However, for this task, NIOTS makes hyperparameters smart search through efficient metaheuristics, highlighting in this context the APMT-MODE. Besides, the PSP is modeled as a MOOP approach providing the designer with a set of high-quality solutions that should contemplate several constraints for SVM/SVR applications. In the NIOTS the PF quality, obtained for each metaheuristic, is measured with the *Inverted Generational Distance* (IGD) metric (Ishibuchi, 2015). Eq. 2 shows as IGD is calculated.

(2) $$IGD\;\left(S,\;P^{*}\right)=\frac{1}{\left|P^{*}\right|}\sum_{\;y\;\in \;P^{*}}dist\left(y,\;S\right)\;\;\;\;\;\;\;\;\;\;\;\;\;\;\;\;\;\;\;\;\;\;\;\;\;\;$$

where $P^{*}\;$ is the true PF, dist(y,S) is the Euclidean distance between $y\;\in \;P^{*}$ from the nearest point in *S*, which is the set of solutions obtained by the metaheuristics, and $|P^{*}|$ the cardinality of true PF. The IGD measures the convergence and spread of the *S* set and the lower the IGD the better is the *S* set.

Taking into account that in practice we do not have an optimal PF, in the NIOTS each metaheuristic generates several non-dominant sets that are used for obtaining a general non-dominant set of solutions by achieving the union of these partial sets, that we adopt as the true PF for PSP [6] [7].

## Declaration of Competing Interest

The authors declare that they have no known competing financial interests or personal relationships that could have appeared to influence the work reported in this paper.
